# Construction of a predictive model for in-hospital mortality in patients with acute myocardial infarction complicated with cardiogenic shock

**DOI:** 10.3389/fcvm.2025.1614183

**Published:** 2025-10-16

**Authors:** Deqiang Yuan, Jun Qian, Hao Lin, Jiapeng Chu, Guoqi Zhu, Fei Chen, Xuebo Liu

**Affiliations:** Department of Cardiology, Tongji Hospital, School of Medicine, Tongji University, Shanghai, China

**Keywords:** acute myocardial infarction, cardiogenic shock, in-hospital mortality, risk prediction model, nomogram

## Abstract

**Background and objective:**

Acute myocardial infarction (AMI) complicated by cardiogenic shock (CS) carries a substantial risk of morbidity and mortality. However, a validated clinical prediction model for in-hospital mortality in these patients is still lacking. This study seeks to develop and validate a mortality risk prediction tool to assist clinicians in early identification of high-risk patients and guide personalized therapeutic interventions.

**Methods:**

We conducted a retrospective analysis of clinical data from 1,419 patients diagnosed with AMI. Of these, 150 patients with AMI complicated by CS were enrolled. Participants were randomly assigned to a training group (70%) or a testing group (30%). Following logistic regression analysis, variables were selected using LASSO regression. Seven candidate predictors were selected for inclusion in the final nomogram model. Model performance was assessed through the area under the receiver operating characteristic curve (AUC), decision curve analysis (DCA), and calibration curves.

**Results:**

A total of 150 patients with AMI complicated by CS were included in the study. In-hospital mortality occurred in 41 patients (27.33%). Eleven variables, including age, smokers, and left ventricular ejection fraction (LVEF), were identified as potential predictors of in-hospital mortality. The final nomogram incorporated the following independent predictors: age, LVEF, creatine kinase-MB (CK-MB), high-sensitivity C-reactive protein (Hs-CRP), β-blocker use, angiotensin-converting enzyme inhibitor/angiotensin receptor blocker (ACEI/ARB) use, and statin use. During internal validation, the nomogram demonstrated AUC values of 0.941 in the training sets and 0.981 in the testing sets. Both calibration curves and DCA showed excellent agreement between predicted probabilities and observed outcomes.

**Conclusion:**

This study developed and internally validated a clinically applicable prediction model and nomogram for assessing the risk of in-hospital mortality among patients with AMI complicated by CS. The results offer readily applicable insights to guide clinical practitioners in implementing timely, personalized patient management strategies.

## Introduction

Acute myocardial infarction (AMI) complicated by cardiogenic shock (CS) presents a major clinical challenge, characterized by high morbidity and mortality rates (1, 2). Accurate prediction of in-hospital mortality in these patients is critical for implementing timely and appropriate management strategies. Although existing studies have identified several risk factors for AMI patients with CS, there remains a lack of readily applicable predictive models for clinical use (3–5). A thorough understanding of mortality-associated risk factors in this population is essential to establish a foundation for informed clinical decision-making (6, 7).

In recent years, researchers have increasingly focused on predictive modeling to identify patients at increased risk of mortality (8–10). These models integrate diverse patient-specific variables to generate accurate and timely prognostic estimates. Consequently, healthcare practitioners can optimize interventions, resource allocation, and patient care strategies (11). Recent advances in predictive modeling have created new opportunities for risk stratification and mortality prediction across various medical conditions. While several validated prognostic scores exist for cardiogenic shock [e.g., IABP-SHOCK II (6, 12, 13), CardShock (14)], these tools often incorporate variables requiring invasive procedures (e.g., arterial lactate, cardiac power index) or specialized laboratory tests not universally available during initial emergency assessment. Furthermore, their development cohorts included mixed etiologies of CS, with only subsets specifically focused on AMI-CS. There remains a need for a practical tool leveraging routinely available early data to facilitate rapid risk stratification specifically in AMI-CS patients at the point of care. To address this gap, we developed a practical and comprehensive model specifically designed to predict in-hospital mortality in this patient population.

The primary objective of this study was to aid healthcare providers in the early identification of high-risk patients to support informed decision-making regarding treatment selection and resource allocation. By developing and validating a clinically applicable model for predicting in-hospital mortality among patients with AMI complicated by CS, we aimed to provide clinicians with a practical prognostic tool to guide individualized patient management. Our approach leveraged established statistical methodologies to construct a model for predicting in-hospital mortality in this high-risk cohort.

## Methods

### Patients

**Figure 1:**
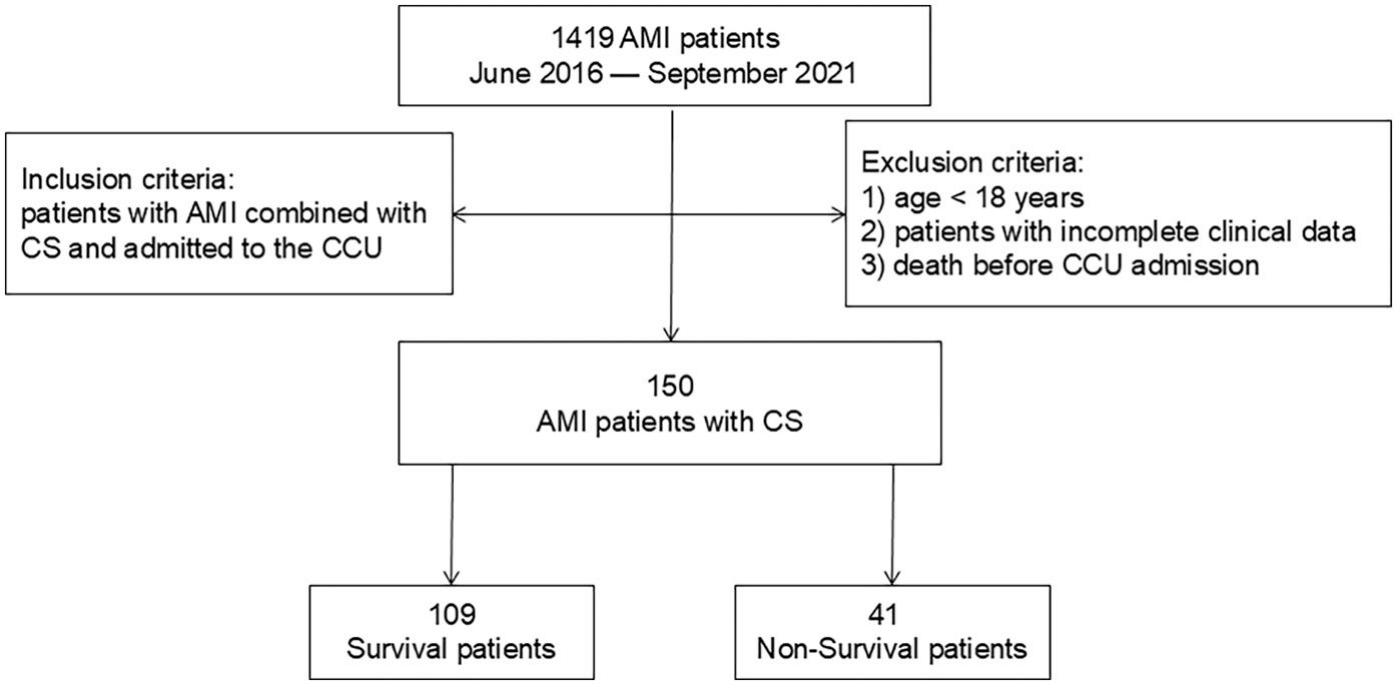
Study workflow.

Ethical approval for this retrospective study was obtained, with a waiver of informed consent requirement granted. We retrospectively reviewed the clinical database from the Department of Cardiology at Shanghai Tongji Hospital, including data for 1,419 patients diagnosed with AMI between June 2016 and September 2021. Inclusion criteria were: (1) diagnosis of AMI complicated by CS; (2) admission to the cardiac intensive care unit (CCU). CS was defined as a sustained systolic blood pressure ≤90 mmHg for ≥30 min (15). Exclusion criteria were: (1) age <18 years; (2) incomplete medical records; (3) death before CCU admission. Ultimately, 150 patients with AMI complicated by CS were included. The patient selection flowchart is presented in Figure 1.

### Variable selection and model construction

Clinical data were collected anonymously from electronic medical records within 24 h of admission. Data included demographic characteristics, comorbidities, presenting symptoms, admission vital signs, laboratory results (hematological, biochemical, inflammatory, and coagulation markers), and medication details. Use of β-blockers, angiotensin-converting enzyme inhibitors and angiotensin 2 receptor blockers (ACEI/ARB), and statins was defined as documented administration at any point during the index hospitalization prior to the outcome event (in-hospital death) or discharge. All laboratory values used in model development, including CK-MB and hs-CRP, were the first measurements obtained upon hospital admission (i.e., within 2 h of emergency department arrival or direct admission). Peak values during hospitalization were not utilized for model building, ensuring the model's applicability for early prognostication at the time of initial patient assessment. Patients were followed until hospital discharge, with in-hospital mortality as the primary endpoint. The cohort of 150 AMI-CS patients was randomly divided into a training set (70%, *n* = 105) for model development and a testing set (30%, *n* = 45) for internal validation. Stratified random sampling based on in-hospital mortality status was performed using R software version 4.0.2 (R Foundation for Statistical Computing) with the “createDataPartition()” function (“caret” package v6.0-94). A fixed random seed (1,234) ensured reproducibility. Mortality rates were comparable across sets: training set (29 deaths, 27.6%), testing set (12 deaths, 26.7%), and overall cohort (27.3%). This allocation balanced model stability needs with independent validation requirements.

### Statistical analysis

Statistical analyses were performed using R software. Normally distributed continuous variables are presented as mean ± standard deviation and were compared using independent *t*-test (two groups), with *post-hoc* least significant difference (LSD) testing. Non-normally distributed continuous variables are expressed as median (interquartile range) and compared using the Mann–Whitney *U* test. Categorical variables are reported as frequency (percentage) and compared using *χ*² tests. Variables with <10% missing values were imputed using the mice package in R.

Following the stratified random sampling described previously, the training set was used for model development via logistic regression analysis. LASSO regression was implemented via “glmnet” package in R with: (a) *λ* determination: 10-fold cross-validation minimizing binomial deviance; (b) Threshold criterion: Variables retained at *λ* = *λ*min (minimum deviance criterion); (c) Standardization: Continuous variables scaled to unit variance; (d) Convergence: 10^−4^ tolerance threshold over 1,000 iterations. The 10-fold cross-validation method was applied to verify the stability and reliability of the LASSO regression results. A nomogram was constructed to visualize the final prediction model. Model performance was assessed in both sets using: (a) the area under the receiver operating characteristic curve (AUC) for discrimination; (b) calibration curves for goodness-of-fit; and (c) decision curve analysis (DCA) for clinical utility.

## Results

### Clinical characteristics

The final cohort comprised 150 patients with a mean age of 68.76 years and 80% male representation. Among these, 109 individuals survived with mean age of 66.14 years and 81.65% being male, while 41 individuals did not survive with mean age of 72.44 years and 75.61% being male; survivors were significantly younger than non-survivors (*p* = 0.002). Baseline characteristics differed significantly between groups in: age, smokers, and left ventricular ejection fraction (LVEF), high-sensitivity cardiac troponin I (Hs-cTnI), creatine kinase myocardial band (CK-MB), creatinine (Cr), high-sensitivity C-reactive protein (Hs-CRP), N-terminal fragment brain natriuretic peptides (NT-pro-BNP), β-blocker use, ACEI/ARB use, and statin use (*p* < 0.05). The percentage of smokers among survivors was markedly greater than that among non-survivors (66.06% vs. 24.39%, *p* < 0.001), as was the utilization of β-blockers (61.47% vs. 9.76%, *p* < 0.001), ACEI/ARBs (65.14% vs. 12.20%, *p* < 0.001), and statins (95.41% vs. 29.27%, *p* < 0.001). Survivors exhibited a notably higher LVEF than non-survivors (53.06% vs. 48.71%, *p* = 0.021). Conversely, survivors had significantly lower levels of Hs-cTnI (0.11 vs. 3.47, *p* = 0.003), CK-MB (5.90 vs. 24.20, *p* = 0.001), Cr (101.10 vs. 132.29, *p* = 0.006), Hs-CRP (30.71 vs. 47.86, *p* = 0.041), and NT-pro-BNP (1,314.00 vs. 3,830.50 pg/ml, *p* = 0.016) compared to non-survivors. Parameters such as sex, hypertension, diabetes mellitus (DM), stroke history, prior myocardial infarction (MI), history of percutaneous coronary intervention (PCI), and certain laboratory parameters such as blood urea nitrogen (BUN), estimated glomerular filtration rate (eGFR), D-dimer, fasting glucose, hemoglobin A1c (HbA1c), total cholesterol (TC), low-density lipoprotein cholesterol (LDL-C), hemoglobin (Hb), and body mass index (BMI) did not exhibit significant differences between the two groups (all *p* > 0.05). Detailed patient characteristics for survivors and non-survivors are presented in Table 1.

**Table 1:** Patient demographics and clinical features.

Variables	Total (*n* = 150)	Survival		*P* value
		Yes (*n* = 109)	No (*n* = 41)	
Demographic characteristics				
Age, y	67.86 ± 11.39	66.14 ± 10.69	72.44 ± 12.03	**0** **.** **002**
Male, *n* (%)	120 (80.00%)	89 (81.65%)	31 (75.61%)	0.410
Comorbidities				
Hypertension, *n* (%)	88 (58.67%)	65 (59.63%)	23 (56.10%)	0.695
DM, *n* (%)	52 (34.67%)	40 (36.70%)	12 (29.27%)	0.394
Smokers, *n* (%)	82 (54.67%)	72 (66.06%)	10 (24.39%)	**<0** **.** **001**
Stroke, *n* (%)	30 (20.00%)	20 (18.35%)	10 (24.39%)	0.410
Prior MI, *n* (%)	3 (2.00%)	1 (0.92%)	2 (4.88%)	0.181
PCI History, *n* (%)	8 (5.33%)	4 (3.67%)	4 (9.76%)	0.215
Laboratory examinations				
LVEF, %	51.8 ± 10.32	53.06 ± 11.38	48.71 ± 5.70	**0** **.** **021**
Hs-cTnI (ng/ml)	0.26 (0.03–7.50)	0.11 (0.02–3.91)	3.47 (1.07–14.10)	**0** **.** **003**
CK-MB (ng/ml)	9.90 (2.32–34.40)	5.90 (2.10–34.40)	24.20 (5.50–84.60)	**0** **.** **001**
BUN (mmol/L)	7.00 (5.60–10.35)	7.00 (5.40–10.40)	6.90 (5.90–9.90)	0.542
Cr (μmol/L)	109.63 ± 62.48	101.10 ± 57.23	132.29 ± 70.54	**0** **.** **006**
eGFR (ml/min/1.73 m^2^)	79.75 ± 63.31	83.89 ± 69.64	68.73 ± 40.78	0.192
D-dimer (μg/ml)	0.98 (0.34–2.19)	0.68 (0.32–2.19)	1.70 (0.81–2.91)	0.115
Fasting Glucose (mmol/L)	9.67 ± 4.46	9.29 ± 4.63	10.68 ± 3.85	0.090
HbA1c (%)	7.11 ± 1.63	7.12 ± 1.86	7.10 ± 0.74	0.935
TC (mmol/L)	4.61 ± 1.13	4.70 ± 1.22	4.39 ± 0.80	0.129
LDL-C (mmol/L)	3.13 ± 0.83	3.20 ± 0.90	2.93 ± 0.58	0.080
Hs-CRP (mg/L)	18.80 (5.99–36.22)	30.71 ± 43.09	47.86 ± 51.02	**0** **.** **041**
NT-pro-BNP (pg/ml)	2,134.00 (565.57–3,830.50)	1,314.00 (454.90–3,830.50)	3,830.50 (3,830.50–5,397.00)	**0** **.** **016**
Hb (g/L)	138.69 ± 18.76	139.60 ± 18.26	136.27 ± 20.05	0.335
BMI	23.66 ± 2.63	23.70 ± 2.81	23.55 ± 2.12	0.750
β-blocker use, *n* (%)	71 (47.33%)	67 (61.47%)	4 (9.76%)	**<0** **.** **001**
ACEI/ARB use, *n* (%)	76 (50.67%)	71 (65.14%)	5 (12.20%)	**<0** **.** **001**
Statin use, *n* (%)	116 (77.33%)	104 (95.41%)	12 (29.27%)	**<0** **.** **001**

DM, diabetes mellitus; MI, myocardial infarction; PCI, percutaneous coronary intervention; LVEF, left ventricular ejection fraction; Hs-cTnI, high-sensitivity cardiac troponin I; CK-MB, creatine kinase-MB; BUN, blood urea nitrogen; Cr, creatinine; eGFR, estimated glomerular filtration rate; HbA1c, Hemoglobin A1c; TC, total cholesterol; LDL-C, low-density lipoprotein cholesterol; Hs-CRP, hypersensitive C-reactive protein; NT-pro-BNP, N-terminal pro-B-type natriuretic peptide; Hb, hemoglobin; BMI, body mass index; ACEI/ARB, angiotensin converting enzyme inhibitors and angiotensin 2 receptor blockers.

Bold values indicate statistical significance at *p* < 0.05.

### Predictors of in-hospital mortality

In-hospital mortality occurred in 41 patients (27.33%). Univariate logistic regression analyses applied to the 150 patients' cohort reduced 26 initial baseline variables to 11 candidate predictors. These potential predictors included age, smokers, LVEF, Hs-cTnI, CK-MB, Cr, Hs-CRP, NT-pro-BNP, β-blocker use, ACEI/ARB use, and statin use. The results of the univariate logistic regression analyses for these predictors are presented in Table 2. Age [odds ratio (OR) = 1.05; 95% confidence interval (CI), 1.02–1.09; *p* = 0.0033], smokers (OR = 0.17; 95% CI, 0.07–0.37; *p* < 0.001), LVEF (OR = 0.96; 95% CI, 0.92–0.99; *p* = 0.0246), Hs-cTnI (OR = 1.03; 95% CI, 1.01–1.04; *p* = 0.0091), CK-MB (OR = 1.01; 95% CI, 1.00–1.01; *p* = 0.0042), Cr (OR = 1.01; 95% CI, 1.00–1.01; *p* = 0.0154), Hs-CRP (OR = 1.01; 95% CI, 1.00–1.01; *p* = 0.0499), NT-pro-BNP (OR = 1.01; 95% CI, 1.00–1.01; *p* = 0.0248), β-blocker use (OR = 0.07; 95% CI, 0.02–0.20; *p* < 0.001), ACEI/ARB use (OR = 0.07; 95% CI, 0.03–0.21; *p* < 0.001), and statin use (OR = 0.02; 95% CI, 0.01–0.06; *p* < 0.001) emerged as significant predictors of in-hospital mortality. On multivariate logistic analysis, ACEI/ARB use (aOR = 0.17; 95% CI, 0.04–0.84; *p* = 0.029) and statin use (aOR = 0.03; 95% CI, 0.01–0.18; *p* < 0.001) remained independently associated with reduced mortality risk after adjustment.

**Table 2:** Univariate and multivariate variables associated with in-hospital mortality using the logistic models.

Variables	Univariate analysis		Multivariable analysis	
	OR (95% CI)	*P* value	OR (95% CI)	*P* value
Age	1.05 (1.02, 1.09)	0.0033	1.02 (0.97, 1.08)	0.444
Smokers	0.17 (0.07, 0.37)	<0.001	1.28 (0.30–5.42)	0.739
LVEF	0.96 (0.92, 0.99)	0.0246	0.94 (0.88–1.00)	0.059
Hs-cTnI	1.03 (1.01, 1.04)	0.0091	0.99 (0.96–1.03)	0.719
CK-MB	1.01 (1.00, 1.01)	0.0042	1.01 (1.00–1.02)	0.125
Cr	1.01 (1.00, 1.01)	0.0154	1.00 (0.99–1.01)	0.986
Hs-CRP	1.01 (1.00, 1.01)	0.0499	1.01 (1.00–1.02)	0.076
NT-pro-BNP	1.00 (1.00, 1.00)	0.0248	1.00 (1.00–1.00)	0.513
β-blocker use	0.07 (0.02, 0.20)	<0.001	0.39 (0.08–1.78)	0.223
ACEI/ARB use	0.07 (0.03, 0.21)	<0.001	0.17 (0.04–0.84)	0.029
Statin use	0.02 (0.01, 0.06)	<0.001	0.03 (0.01–0.18)	<0.001

LVEF, left ventricular ejection fraction; Hs-cTnI, high-sensitivity cardiac troponin I; CK-MB, creatine kinase-MB; Cr, creatinine; Hs-CRP, hypersensitive C-reactive protein; NT-pro-BNP, N-terminal pro-B-type natriuretic peptide; ACEI, angiotensin-converting enzyme inhibitors; ARB, angiotensin II receptor blockers.

### Development of the predictive nomogram

Based on univariate logistic regression analysis, 11 candidate predictors were initially identified. Subsequently, LASSO regression with 10-fold cross-validation (λ.min criterion) was applied exclusively to these pre-screened variables to mitigate multicollinearity and optimize predictive efficiency. Final predictors for the nomogram were selected through dual criteria: retention of non-zero coefficients in LASSO regression and established clinical relevance per current guidelines. The seven predictors incorporated into the nomogram were: ACEI/ARB use, statin use, age, LVEF, CK-MB, hs-CRP, and β-blocker use. These variables demonstrated both statistical significance and clinical utility in the prediction model. Four variables were excluded: creatinine, NT-proBNP, smoking status, and hs-cTnI. This exclusion resulted from LASSO coefficient shrinkage to zero and considerations of clinical interpretability. The 10-fold cross-validation method verified the stability and reliability of the LASSO regression results, with narrow confidence bands indicating robust model stability (Supplementary Figures S1A,B).

**Figure 2:**
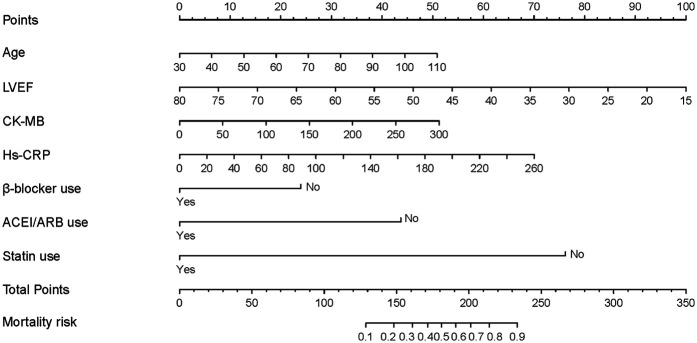
The nomogram to predict in-hospital mortality was created based on 7 significant predictors. *(Branch: 1 means the presence of branch type MB; 0 means the absence of branch type MB).

The resulting nomogram is shown in Figure 2. In this nomogram, a higher total score assigned to each predictor indicates an increased risk of mortality during hospitalization. Each point on the nomogram represents a specific scoring standard or scale. A perpendicular line was drawn for each independent variable to determine the corresponding score based on its value. For instance, an age of 70 equated to 25.1 points, while a lack of statin use equated to 75 points. The cumulative points for all independent variables were calculated for each patient, and their corresponding risk levels for in-hospital mortality in AMI with CS patients were estimated based on the location on the perpendicular line.

### Validation of the predictive nomogram

**Figure 3:**
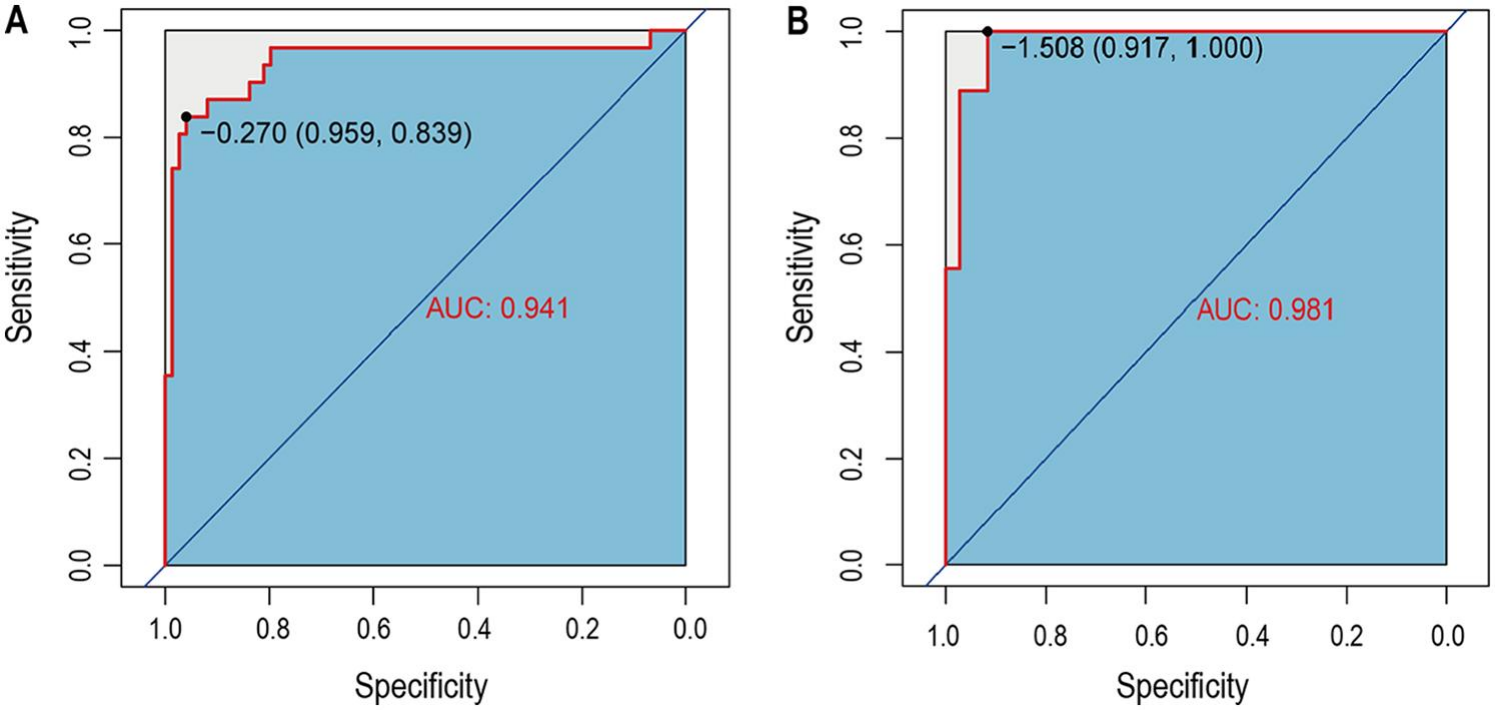
The time-dependent receiver operating characteristic (ROC) curve and area under the ROC curve (AUC). **(A)** The ROC in training set; **(B)** The ROC in testing set.

The nomogram's performance was internally validated using the training and testing cohorts. In the training cohort, the nomogram model achieved an AUC of 0.941 (95% CI, 0.839–0.959) as shown in Figure 3A. In the testing cohort, the AUC was 0.981 (95% CI, 0.917–1.000) (Figure 3B), indicating strong predictive ability for in-hospital mortality in AMI-CS patients. These internal validation results confirmed the model's robust predictive accuracy.

### Assessment of the predictive nomogram

**Figure 4:**
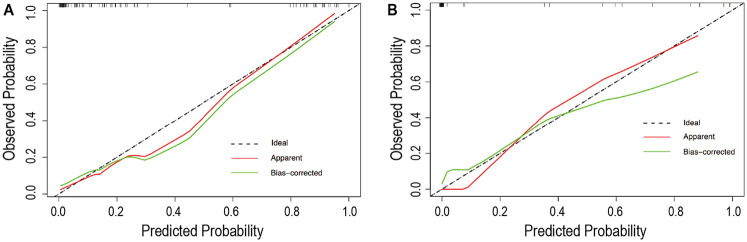
The calibration curves for evaluating the accuracy of the nomogram. **(A)** The calibration curve in training set; **(B)** The calibration curve in testing set.

**Figure 5:**
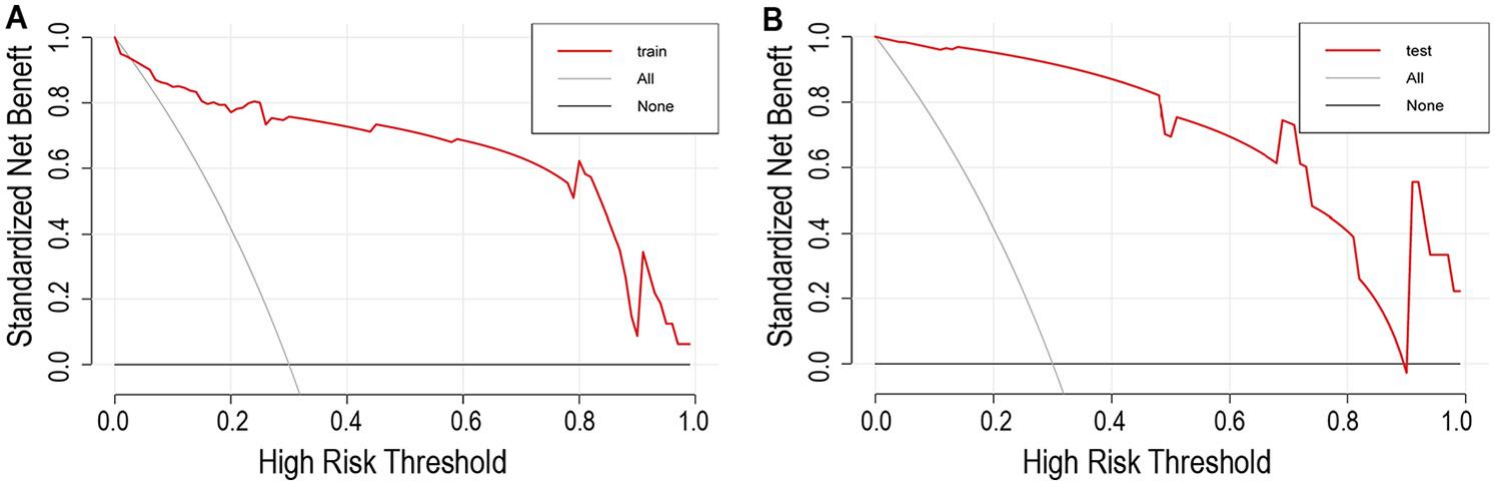
Decision curve analysis for the nomogram and the model with predictors. **(A)** The decision curve in training set; **(B)** The decision curve in testing set. The *y*-axis measures the net benefit. The red line represents the nomogram. The red gray line represents the assumption that all patients have in-hospital mortality. The black line represents the assumption that no patients have in-hospital mortality.

Calibration curves demonstrated good agreement between predicted and observed mortality rates in both the training group (Figure 4A) and the testing group (Figure 4B). Furthermore, the decision curve analysis confirmed the model's clinical utility for practical application, showing a positive net benefit across a wide range of threshold probabilities, as depicted in Figure 5.

## Discussion

This study developed and internally validated a clinical prediction nomogram incorporating key clinical and laboratory predictors to estimate individualized in-hospital mortality risk for patients with AMI complicated by CS. The model demonstrated excellent discrimination, with AUC values of 0.941 in the training cohort and 0.981 in the testing cohort. Calibration and decision curve analyses further confirmed its clinical applicability. This practical tool addresses a critical unmet need in AMI-CS management by enabling early identification of high-risk patients to guide personalized treatment decisions.

CS represents the predominant cause of in-hospital mortality in AMI, contributing to high fatality rates and complex management challenges (15, 16). Our cohort reflected this burden, with 27.3% in-hospital mortality. This high-risk context requires urgent optimization of risk stratification. To address this need, we developed and validated a clinically applicable prediction model using robust statistical methods. Key strengths include its excellent discriminatory accuracy (AUC 0.981 in testing) and immediate clinical implementability, as all incorporated variables are routinely collected during initial assessment without specialized testing. In-hospital mortality in AMI complicated by CS has been extensively studied, with research focusing on identifying prognostic predictors. For instance, a study of 1,333 AMI-CS patients undergoing primary PCI identified post-PCI thrombolysis in myocardial infarction (TIMI) < 3 flow, advanced age, three-vessel disease, and prolonged symptom-to-PCI time as independent predictors of mortality (17). While valuable, this PCI-centric approach did not incorporate readily available admission parameters such as laboratory biomarkers, medication use, or comorbidities that may enhance early risk assessment. Another investigation of 1,102 AMI patients (including 196 CS cases) reported 17.8% overall CS mortality, with 39.3% mortality specifically in the CS subgroup (18). In this cohort, NSTEMI presentation and reduced LVEF were independent mortality predictors—findings consistent with our results regarding age and cardiac function. Our study extends this evidence by demonstrating the prognostic significance of guideline-directed medications (β-blockers, ACEI/ARBs, statins) and inflammatory biomarkers (hs-CRP) in a comprehensive prediction model.

A study of 319 ST-elevation myocardial infarction (STEMI) patients with and CS undergoing PCI reported 61.3% in-hospital mortality (19). Multivariable analysis identified chronic renal insufficiency, post-PCI TIMI score ≤2, hyperglycemia, hyperlactatemia, elevated blood urea nitrogen, reduced Tricuspid Annular Plane Systolic Excursion (TAPSE), and decreased ejection fraction as independent predictors of in-hospital mortality. Unlike these models relying on post-intervention parameters, our approach integrated statistical learning methods for variable selection to develop a prediction model based on admission data. Similarly, a study of 274 STEMI-CS patients (65.3% in-hospital mortality) used multivariable logistic regression to construct a nomogram incorporating sex, admission glucose, intra-aortic balloon pump (IABP) use, no-reflow phenomenon, and post-PCI ejection fraction (20). While providing valuable insights, their predictors primarily required procedural data, limiting early risk assessment capability. Conversely, our model utilizes routinely available admission variables—laboratory biomarkers and medication profiles—enabling immediate risk stratification without specialized interventions. This enhances practical utility across diverse healthcare settings. Nomograms offer visual quantification of predicted mortality risk; our instrument prioritizes clinically accessible variables to maximize adoption potential. Validation confirmed the model's robust performance, allowing physicians to calculate personalized mortality risk, stratify patients into risk categories, identify high-risk cases, and tailor early interventions accordingly. Non-survivors, on average, 6 years older than survivors in our study. This finding aligns with Damluji et al. (21), who reported increasing age as an independent predictor of in-hospital mortality in STEMI-CS patients. The observed association likely reflects age-related declines in physiological reserve and elevated vulnerability to various complications in the elderly. Notably, although smoking is an established cardiovascular risk factor (22), our study found a significantly higher percentage of survivors were smokers in comparison to those hospitalized patients who died. This apparent paradox may be explained by the significantly younger age and potentially preserved functional status in smoking patients—factors independently associated with improved outcomes in critical illness. Future multi-center studies with larger cohorts should validate this counterintuitive association.

IABP-SHOCK II and CardShock are also important tools for predicting the mortality of CS. Our model demonstrated significantly higher discriminative accuracy (AUC 0.94) compared to the IABP-SHOCK II (AUC 0.77) (12) and CardShock (AUC 0.79) (14) scores within our cohort. We attribute this difference primarily to two factors: (1) Temporal alignment of predictors: Our model incorporates key variables reflecting the severity of the acute ischemic insult (CK-MB) and systemic inflammation (hs-CRP), along with early treatment decisions, which may more directly capture the pathophysiology driving mortality in AMI-CS. In contrast, scores like IABP-SHOCK II rely heavily on variables like lactate and creatinine, which reflect downstream organ hypoperfusion but may exhibit greater variability in timing and measurement; (2) Cohort specificity: While IABP-SHOCK II and CardShock are valuable general CS scores, our model was specifically developed and tuned for AMI-CS. The inclusion of AMI-specific markers (CK-MB) and treatments likely enhances its performance in this subset. However, we acknowledge that our higher AUC must be interpreted cautiously, as it stems from internal validation and reflects performance within the same cohort used for development. External validation is essential to confirm whether this performance gap persists. The choice of prognostic tool should consider the clinical context. The IABP-SHOCK II and CardShock scores remain valuable, particularly for initial triage in undifferentiated shock or settings lacking immediate AMI-specific biomarkers. Our nomogram offers an alternative optimized for AMI-CS patients once key initial results (LVEF, CK-MB, hs-CRP) are available, potentially aiding decisions regarding escalation of mechanical circulatory support or palliative care consultation later in the ICU course. Therefore, our tool complements rather than replaces established scores, especially in resource-rich settings.

Beyond traditional clinical scores, machine learning and artificial intelligence techniques are increasingly applied to predict outcomes in AMI and CS (23). Recent studies have leveraged complex algorithms and high-dimensional data to achieve high predictive accuracy. Recent advances in machine learning have demonstrated the potential to improve prognostic accuracy, as seen in models by Hu et al. (24) and Zhang et al. (25) achieving AUCs >0.80. Our proposed model builds upon these foundations by incorporating novel biomarkers and dynamic data integration, aiming to further enhance predictive performance. While demonstrating impressive performance, these models often function as “black boxes”, require extensive computational resources, and depend on data not routinely available at initial presentation. Our study aims to bridge this gap by developing a transparent, interpretable nomogram based on readily available early clinical and laboratory parameters, offering a practical tool for immediate bedside use.

Our study further identified LVEF, CK-MB, and Hs-CRP as significant predictors of in-hospital mortality in AMI-CS patients. Physiologically, LVEF quantifies left ventricular systolic function, typically falls within the normal range of 50%–70%, with values <50% indicating cardiac dysfunction. This aligns with established evidence linking reduced LVEF to increased mortality risk, including post-ablation ventricular tachycardia cohorts (26). Similarly, CK-MB—a cardiac-specific enzyme—correlates with myocardial injury extent. Elevated CK-MB levels portend heightened in-hospital death risk, as demonstrated in pulmonary embolism studies (27). Notably, Hs-CRP provides high-sensitivity quantification of systemic inflammation. Its elevation independently predicts cardiovascular risk and mortality (28), with our results extending this association to AMI-CS. Collectively, these findings reinforce the pathophysiological and prognostic roles of ventricular dysfunction (LVEF), myocardial necrosis (CK-MB), and systemic inflammation (hs-CRP) in AMI-CS mortality. A notable finding was the inclusion of β-blocker, ACEI/ARB, and statin use as independent predictors of in-hospital mortality in AMI-CS patients. Current evidence underscores immediate revascularization of infarct-related coronary arteries as the standard of care for CS secondary to AMI, as substantiated by randomized clinical trials (15, 29). Findings from the predictive model indicated that the utilization of β-blockers, ACEI/ARBs, and statins was linked to a reduced risk of in-hospital mortality, conveying significant implications for clinical management practices. This underscores the critical importance of guideline-directed medical therapy initiation post-revascularization. Notably, β-blockers are contraindicated in acute CS due to negative inotropic effects (16). Nonetheless, the predictive model suggests that as AMI progresses and hemodynamic stability is achieved, β-blocker therapy may enhance prognosis and mitigate in-hospital mortality in AMI-CS patients. The beneficial outcomes attributed to β-blockers, including antagonism of catecholamine adrenergic neurotransmitters, antihypertensive peculiarity, anti-ischemic role, and antiarrhythmic benefits, may underpin the observed prognostic improvements (30). Similarly, ACEI/ARBs mitigate ventricular remodeling, while statins exert pleiotropic cardioprotective effects via lipid-lowering, anti-inflammatory, and plaque-stabilizing properties (31, 32). Future studies should elucidate optimal timing and mechanisms of statins, β-blockers, and ACEI/ARBs therapy in AMI-CS.

While the calibration curve analysis demonstrated strong overall concordance between predicted and observed outcomes in both training and testing sets as showed in Figure 4, we note a modest decrease in calibration performance following model adjustment. This phenomenon primarily stems from two interrelated factors inherent to clinical prediction modeling: (1) The bias-variance tradeoff in regularization: Our LASSO regularization approach, while effectively reducing overfitting by shrinking coefficients of less important predictors, inherently introduces a small amount of bias. This regularization effect is most pronounced in predictors with weaker associations, where coefficient shrinkage creates a conservative estimation bias. This represents an intentional compromise where we accept modest calibration degradation in exchange for improved model generalizability and stability; (2) Finite-sample effects in validation: Our validation cohort (*n* = 45) represents a modest sample size relative to model complexity. When applying the training-derived model parameters to this finite independent sample, we observe the expected phenomenon described by Van Calster et al. (2016) (33): validation calibration curves typically exhibit slightly greater deviation from perfect alignment than training curves due to sampling variability. This effect is particularly noticeable in the extreme probability ranges (<20% and >80%) where event counts are sparse. Importantly, despite this modest adjustment effect, the model maintains excellent clinical utility as evidenced by: persistent high discrimination (AUC 0.981 in testing), favorable decision curve analysis across clinical thresholds, and close alignment with the ideal calibration line. Ongoing calibration refinement is valuable, the published nomogram should be periodically recalibrated during implementation as recommended by the TRIPOD guidelines (34), particularly when applied to populations with different case-mix characteristics.

This study has several limitations. First, it is important to acknowledge that this study adopts a retrospective design and is characterized by a small sample size, which restricts the scientific robustness of the findings due to potential unaccounted confounding variables such as economic status and educational background. Nevertheless, given the inherent challenges in carrying out randomized controlled trials (RCTs) with AMI patients, our study still offers valuable clinical insights. Although our model demonstrated excellent performance in internal validation, the overall cohort size (*n* = 150) and, crucially, the number of in-hospital mortality events (*n* = 41) were relatively small. We acknowledge that the single-center, retrospective nature of our study and the modest sample size are significant limitations that raise valid concerns about potential overfitting and, crucially, the generalizability of our findings. The high AUC observed in both our optimism-corrected derivation cohort and temporal internal validation cohort, while encouraging, must be interpreted with caution. It is possible that this performance reflects unique characteristics of our patient population, clinical practices, or data collection processes at our CCU. Without external validation in independent, multi-center cohorts with varying patient demographics, clinical protocols, and data quality, the true generalizability and real-world performance of our nomogram remain uncertain. This is a critical limitation inherent to our study design. External validation in larger, multi-center prospective cohorts is imperative before this nomogram can be widely adopted in clinical practice. Furthermore, the retrospective design introduces potential biases (e.g., selection bias, information bias) that could influence the model's performance estimates. While our internal validation strategies help mitigate overfitting, they do not account for these broader biases or external factors. Second, gender was not an independent predictor in our model, its potential indirect effects merit consideration. The selected biomarkers (Hs-CRP) and treatments (β-blockers) may have gender-dimorphic effects (35, 36). Future studies should explicitly test whether risk stratification thresholds require gender-specific adjustment. We attempted to incorporate gender into the prediction model, The result indicated that after including gender in the prediction model, the AUC value decreased instead (ΔAUC = −0.085), suggesting that the predictive performance of the model has declined (Supplementary Figures S2A,B). Third, the gender imbalance (80% male) in our cohort, while reflective of AMI-CS epidemiology, limits our ability to fully characterize gender-specific risk patterns. External validation should prioritize cohorts with larger female representation to evaluate potential gender-based calibration differences. Finally, the retrospective nature limited capture of dynamic parameters like serial lactates or vasopressor dosing, preventing Society for Cardiovascular Angiography and Intervention (SCAI) shock classification. Neurological status documentation was heterogeneous. Future models should integrate these standardized elements. Although formal SCAI staging was unavailable, the strong performance of LVEF and Hs-CRP in our model aligns with their known roles in shock pathophysiology. LVEF directly measures cardiac dysfunction (37), while Hs-CRP integrates ischemic injury and systemic inflammation (38)-both central to SCAI's conceptual framework. This suggests our model captures essential biological severity.

A key consideration raised regarding our model is the inclusion of in-hospital medication use (β-blockers, ACEI/ARBs, statins). While these variables were statistically selected by LASSO and contributed to the high discriminative ability of the original model, their incorporation introduces complexity regarding timing and potential immortal time bias. In our CCU, initiation of these therapies often occurred beyond the immediate hyper-acute phase of CS. Consequently, their presence in the model may partly reflect treatment decisions made after initial stabilization or even after patients have survived a critical period, rather than purely baseline risk. This partly limits the model's applicability for immediate (e.g., first hour) bedside risk stratification at the moment of CS diagnosis. Therefore, we performed a sensitivity analysis by rebuilding the prediction model using LASSO regression excluding the three medication variables (β-blocker, ACEI/ARB, statin) (Supplementary Figures S3A,B). The simplified model retained age, LVEF, CK-MB, and hs-CRP as predictors. The AUC of this simplified model was 0.744 in the training set and 0.793 in the internal validation set (Supplementary Figures 3C–E). Compared to 0.941 and 0.981 for the original model, after removing these three medication indicators, the predictive efficacy significantly decreased. It is indicated that these three medication indicators are of great significance in the predictive model. In a way, this simplified model may be more appropriate for the intended purpose of early risk assessment. The original model including medications might be more relevant for prognostication later in the hospitalization course (e.g., after 24–48 h) when treatment decisions have been initiated and documented.

In conclusion, this study developed and validated a clinically applicable nomogram using rigorous statistical modeling to predict in-hospital mortality in patients with AMI complicated by CS. The model incorporates routinely available clinical predictors—including laboratory biomarkers and medication use—enabling frontline clinicians to identify high-risk patients at admission, tailor early interventions, and optimize resource allocation. This tool addresses a critical gap in AMI-CS risk stratification and may improve outcomes through timely, personalized management.

## Data Availability

The raw data supporting the conclusions of this article will be made available by the authors, without undue reservation.
