# Supplementary material for: Construction of a predictive model for in-hospital mortality in patients with acute myocardial infarction complicated with cardiogenic shock
Source: Front Cardiovasc Med. 2025 Oct 16;12:1614183. doi: 10.3389/fcvm.2025.1614183 (PMC12571792; doi:10.3389/fcvm.2025.1614183)
Supplement: Supplementary file 1 [file Datasheet1.pdf]

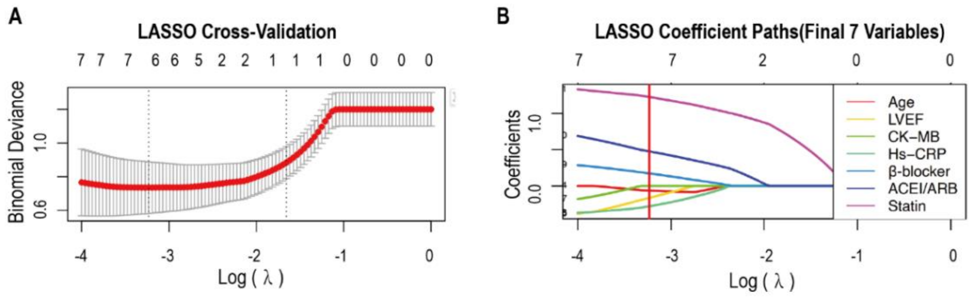

Supplementary Figure 1: Cross-Validation for LASSO Regression. A) Deviance plot showing mean cross-validated error (red dots) ± 1 SD (error bars) across log(λ) values. The vertical dashed lines indicate λ_min (left) and λ_1se (right). The cross-validation error curve demonstrates: stable minimum deviance plateau between log(λ) = -3.5 to -2.5, clear "elbow" at λ_1se where error begins increasing substantially, narrow confidence bands indicating robust model stability; B) Coefficient paths demonstrating how variable coefficients shrink as λ increases. The vertical line at λ_min shows the final selected model with 7 non-zero coefficients.

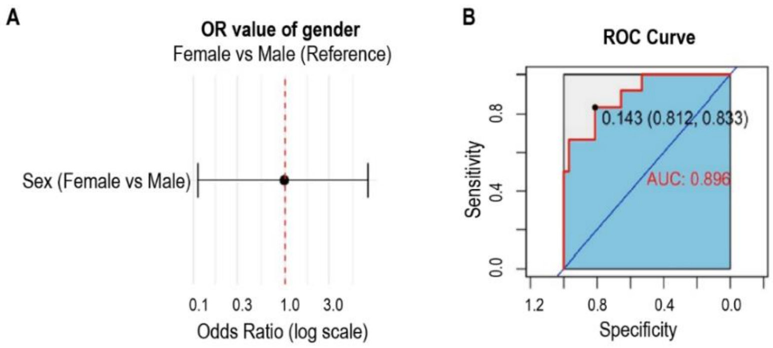

Supplementary Figure 2: Gender OR values and predictive model for the inclusion of genders. A) Added gender to original 7 predictive factors, and the OR value and 95% confidence interval of gender were calculated, showed non-significant association (OR=0.976, 95% CI: 0.112-8.015); B) after including gender in the prediction model, the AUC value = 0.896 decreased instead (ΔAUC=-0.085), suggesting that the predictive performance of the model has declined.

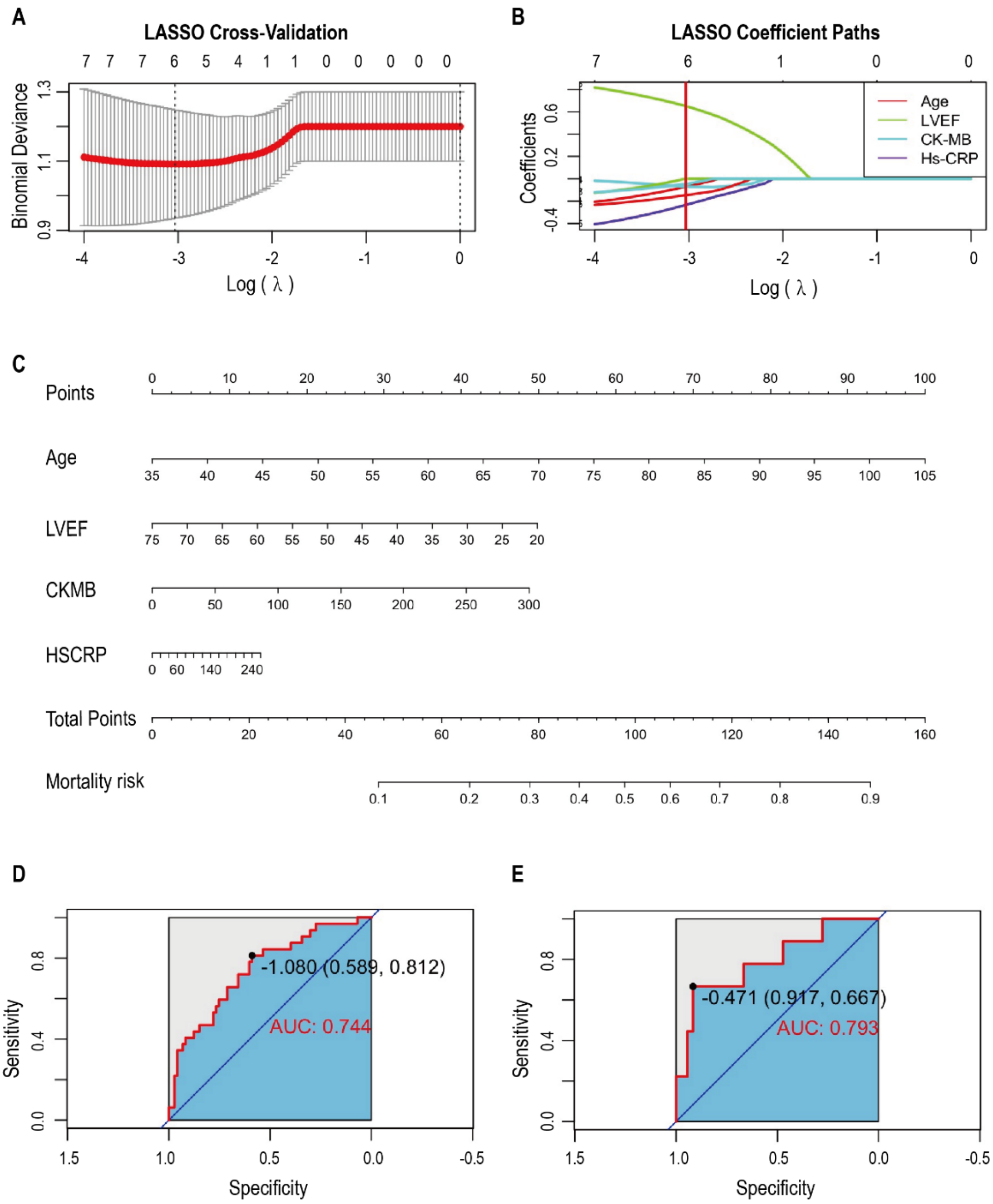

Supplementary Figure 3: Construction of the predictive model after excluding the medication indicators and verification using the ROC curve. A-B) Cross-Validation and Coefficient paths for LASSO Regression, 4 predictive factors (age, LVEF, CK-MB, and hs-CRP) were identified after excluding the medication indicators from the original 7 predictive indicators; C) The nomogram to predict in-hospital mortality was created based on 7 original indicators exclude 3 indicators related to medication use. *(Branch: 1 means the presence of branch type MB; 0 means the absence of branch type MB); D-E) ROC curve and AUC of the simplified model retained age, LVEF, CK-MB, and hs-CRP as predictors. D: The ROC in training set, E: The ROC in testing set.
